# A database of the global distribution of alien macrofungi

**DOI:** 10.3897/BDJ.8.e51459

**Published:** 2020-04-01

**Authors:** Miguel Monteiro, Luís Reino, Anna Schertler, Franz Essl, Rui Figueira, Maria Teresa Ferreira, César Capinha

**Affiliations:** 1 CIBIO/InBIO, Centro de Investigação em Biodiversidade e Recursos Genéticos, Universidade do Porto, Porto, Portugal CIBIO/InBIO, Centro de Investigação em Biodiversidade e Recursos Genéticos, Universidade do Porto Porto Portugal; 2 CIBIO/InBIO, Centro de Investigação em Biodiversidade e Recursos Genéticos, Instituto Superior de Agronomia, Universidade de Lisboa, Lisboa, Portugal CIBIO/InBIO, Centro de Investigação em Biodiversidade e Recursos Genéticos, Instituto Superior de Agronomia, Universidade de Lisboa Lisboa Portugal; 3 Centro de Estudos Florestais, Instituto Superior de Agronomia, Universidade de Lisboa, Lisboa, Portugal Centro de Estudos Florestais, Instituto Superior de Agronomia, Universidade de Lisboa Lisboa Portugal; 4 Division of Conservation Biology, Vegetation Ecology and Landscape Ecology, Department of Botany and Biodiversity Research, University of Vienna, Vienna, Austria Division of Conservation Biology, Vegetation Ecology and Landscape Ecology, Department of Botany and Biodiversity Research, University of Vienna Vienna Austria; 5 LEAF-Linking Landscape, Environment, Agriculture and Food, Instituto Superior de Agronomia, Universidade de Lisboa, Lisboa, Portugal LEAF-Linking Landscape, Environment, Agriculture and Food, Instituto Superior de Agronomia, Universidade de Lisboa Lisboa Portugal; 6 Centro de Estudos Geográficos, Instituto de Geografia e Ordenamento do Território - IGOT, Universidade de Lisboa, Lisboa, Portugal Centro de Estudos Geográficos, Instituto de Geografia e Ordenamento do Território - IGOT, Universidade de Lisboa Lisboa Portugal

## Abstract

**Background:**

Human activities are allowing the ever-increasing dispersal of taxa to beyond their native ranges. Understanding the patterns and implications of these distributional changes requires comprehensive information on the geography of introduced species. Current knowledge about the alien distribution of macrofungi is limited taxonomically and temporally, which severely hinders the study of human-mediated distribution changes for this taxonomic group.

**New information:**

Here, we present a database on the global alien distribution of macrofungi species. Data on the distribution of alien macrofungi were searched in a large number of data sources, including scientific publications, grey literature and online databases. The database compiled includes 1966 records (i.e. species x region combinations) representing 2 phyla, 7 classes, 22 orders, 82 families, 207 genera, 648 species and 31 varieties, forms or subspecies. Dates of introduction records range from 1753 to 2018. Each record includes the location where the alien taxon was identified and, when available, the date of first observation, the host taxa or other important information. This database is a major step forward to the understanding of human-mediated changes in the distribution of macrofungal taxa.

## Introduction

In this publication, we present the recently completed Global Alien Macrofungi Database, a database of distribution records of alien macrofungi aggregated from all relevant sources we could identify, namely publications, reports, databases on invasive alien species and citizen science observations. In total, the dataset contains occurrences for nearly 650 alien species, registered in more than 140 countries and sub-national administrative divisions. This represents an increase of nearly 2.5 times the number of alien records and 3.2 times the number of alien species found in the most comprehensive distribution database for alien ectomycorrhizal fungi available prior to our work (Vellinga et al. 2009). The presented database is expected to provide a valuable contribution towards the increasing understanding of the spatial and temporal dynamics of biological invasions worldwide.

## General description

### Purpose

The main goal was to create a comprehensive global repository of distribution records of macrofungi outside their native ranges, as the under-representation of these species in studies of broad-scale invasion patterns reflects a lack of readily available synthesised information about their distribution in the world (Troudet et al. 2017). Macrofungi, i.e. fungi that exhibit macroscopic spore bearing structures, are an artificial group mostly comprised of ectomycorrhizal and saprotroph fungal species. Those are widely missing in alien invasive species databases, such as the CABI Invasive Species Compendium (https://www.cabi.org/ISC; CABI 2019) and Global Invasive Species Database (http://www.iucngisd.org/gisd; IUCN 2019), because their impacts on native biota are hard to assess and remain largely unknown (Desprez-Loustau 2009, Desprez-Loustau et al. 2010,Vizzini et al. 2009). Nevertheless, macrofungi have been massively introduced into new geographic regions particularly as hitchhikers of exotic plants, in infested wood or soil (Desprez-Loustau 2009).

The specific objectives of our work were:

1. To update and expand previous compilations of the global alien distribution of macrofungi, particularly the work of Vellinga et al. (2009), who compiled data on the distribution of alien ectomycorrhizal fungi worldwide. Here, we integrate their data and extend it both taxonomically (i.e. by considering all macrofungi) and temporally (i.e. by including records published more recently).
2. To highlight the relevance of data circulating outside the scientific community and its importance for the comprehensive representation of alien fungal distributions. For compiling the Global Alien Macrofungi Database, a substantial number of alien records were collected from citizen-science-based websites. Often these data sources were the only ones mentioning alien distributions of taxa for given regions, particularly for species best known by the general public, such as the fly agaric (*Amanita muscaria* (L.) Lam.) or the oyster mushroom (*Pleurotus ostreatus* (Jacq.) P. Kumm.). Two notable examples of such online databases of biodiversity observations used in the research process were iNaturalist (https://www.inaturalist.org; iNaturalist 2019) and mushroom observer (https://mushroomobserver.org; Wilson and Hollinger 2019).
3. To provide a detailed representation of the distribution records of alien macrofungi worldwide, which will be pivotal for advancing current knowledge about the spatio-temporal and taxonomic patterns of fungal invasions and establishing a baseline for comparison with new data collected in the future.

## Project description

### Title

A global database of alien macrofungi.

### Personnel

Monteiro, M.; Reino, L.; Schertler, A.; Essl, F.; Figueira, R.; Ferreira, M.T.; Capinha, C.

### Study area description

Countries from all continents except Antarctica and the first-order administrative divisions of the six largest countries in the world (Australia, Brazil, Canada, China, Russia and United States).

### Design description

The creation of the “Global Alien Macrofungi Database” followed a two-step approach. First, we performed an exhaustive search for data sources supplying occurrence records of macrofungi. Then, we critically assessed and harmonised the collected data and entered it into a standardised database.

Our search and collation of alien macrofungi records were carried out during the years 2017-2019. For the first step, we analysed the database made available by Vellinga et al. (2009), who collected a total of 770 distribution records of ectomycorrhizal fungi from more than 190 publications. However, given the exclusive focus of the database on ectomycorrhizal fungi and the consequential absence of data on saprotrophic species, it can hardly be assumed that the patterns represented in Vellinga et al. (2009) provide a comprehensive portrayal of the global biogeography of alien macrofungi. Hence, we performed a complementary search for alien saprotroph fungi and searched for new records of alien ectomycorrhizal fungi.

For the second step, all collected records were entered into two different datasets. First, we compiled a taxonomic checklist that accounts for all macrofungi taxa we found to be introduced outside their native range. Secondly, we described the alien occurrences of those taxa by including additional data when available, such as dates of introduction, host information and invasion status (e.g. casual, established) in the invaded regions. Here, each entry corresponded to a single record described as an alien taxon in a specific location. If a taxon in a given locality were reported multiple times by different sources, we merged the information into a single database entry and cited the earliest reference in time reporting the record.

### Funding

This work was funded by the FEDER Funds through the Operational Competitiveness Factors Programme - COMPETE and by National Funds through FCT, I.P. - Foundation for Science and Technology within the scope of the project “PTDC/BIA-EVL/30931/2017- POCI-01-0145-FEDER-030931”. Miguel Monteiro was funded by a PhD fellowship SFRH/BD/119170/2016. César Capinha and Luís Reino were funded by National Funds through FCT, I.P., under the programme of ‘Stimulus of Scientific Employment – Individual Support’ within contracts 'CEECIND/02037/2017' and ‘CEECIND/00445/2017’, respectively. Franz Essl and Anna Schertler received funding by the Austrian Science Foundation FWF (grant 3757-B29).

## Sampling methods

### Study extent

We built our database by compiling occurrences of introduced macrofungal species based on an exhaustive search in published and unpublished sources. Data were extracted from peer-reviewed articles, scientific and technical reports, books and book chapters, alien species databases and online citizen-science repositories. Finally, we also approached selected mycologists via email. These experts were contacted and asked if they were aware of records of alien macrofungi or of data resources other than the ones we identified through online searches.

### Sampling description

The data collection process consisted of three different procedures, as is explained below.

**Identifying and obtaining relevant records from publications**

During the search process, we initially looked for records in broader introduced taxa databases, such as the ones for Delivering Alien Invasive Species Inventories for Europe (Hulme et al. 2019), the Global Register of Introduced and Invasive Species (Pagad et al. 2019) and the European Alien Species Information Network (Katsanevakis et al. 2019). In addition, we used general-purpose search engines (i.e. Google) and scientific search engines (Google Scholar, Science Direct and JSTOR) to gather more information from relevant literature. We entered key terms related to fungal invasions in different languages including English, German, French, Spanish and Portuguese. The terms used were ‘introduced´, ’invasive’, ‘established’, ‘alien’, ‘non-native’ and ‘exotic’, which were combined with fungal taxonomic terms, ranging from a generic and higher denomination (e.g. ‘fungi’, ‘macromycetes’, ‘basidiomycota’) to a more specific designation, such as the scientific name (e.g. *Amanita muscaria (L.) Lam., Amanita phalloides Secr.*) or a common name (e.g. fly agaric, death cap). For each combination, we repeated the searches by adding the name of one continent or country, until all continents and countries were being considered. As examples, final search terms would be like ‘European alien fungi’, ‘introduced basidiomycota in United States’ or ‘introduced Amanita muscaria + South America’.

**Cross-checking of alien status**

For each record, we assessed the reliability of the alien status given by the original data sources. Records collected from sources explicitly dealing with alien taxa (e.g. Vellinga et al. 2009), retained the nativity status given by the data. These statuses corresponded either to ‘alien’ or to ‘cryptogenic’ (*sensu* Essl et al. 2018). Records collected from non-specialised sources (e.g. species checklists not considering nativity, grey literature and citizen-science data) were cross-checked against biogeographical information available in scientific literature or with mycologists. Cases where the records referred to regions outside known native ranges, were coded as ‘alien’. Cases in clear biogeographical conflict with known native ranges were not considered for inclusion in our database. Finally, cases where the native or alien status was not possible to identify unambiguously were also not considered.

**Occurrence data entry**

To be included in our database, records had to meet specific criteria regarding taxonomy and locality description. First, a record must describe a macrofungal species having sporocarps of at least 2 mm in size, irrespective of phylogenetic placement (Senn-Irlet et al. 2007). As this was not always clear, we had to double-check our data with relevant fungal literature to be sure that the families or even the orders of the referred species were cited as part of the macroscopic fungi checklists. We also had to be certain that the records were identified at least to the species level, as a way of knowing that all contemplated species were, in fact, alien organisms in the non-native places. Furthermore, the records had to be accompanied by geospatial coordinates or, at minimum, an unambiguous textual designation of location level reference (e.g. region, country and locality). Finally, the record had to represent a fungal species introduced by human activity to a region outside its native range. These tasks were accomplished by the main author (MM) during the years 2017-2019 with the supervision of experts in fungal ecology and biogeography. These experts were also consulted and asked if they were aware of records of alien macrofungi or of data resources other than the ones we identified through online searches.

### Quality control

For the development of the dataset, the records from the original sources were revised by the first author because some of the names of the species were not updated or sometimes misspelled. As a result, some changes at any of the taxonomic ranks (e.g. order, family, genus or species) had to be adopted in conformity with the used nomenclature. Even though, in cases of synonyms, both scientific names were included. The taxonomic revision of scientific names and data checking were performed by using Index Fungorum (Index Fungorum 2019) and Mycobank (Robert et al. 2019). To publish our dataset in the GBIF network, we adjusted our records with the Darwin Core specifications (Wieczorek et al. 2012).

## Geographic coverage

### Description

**Figure 1.**
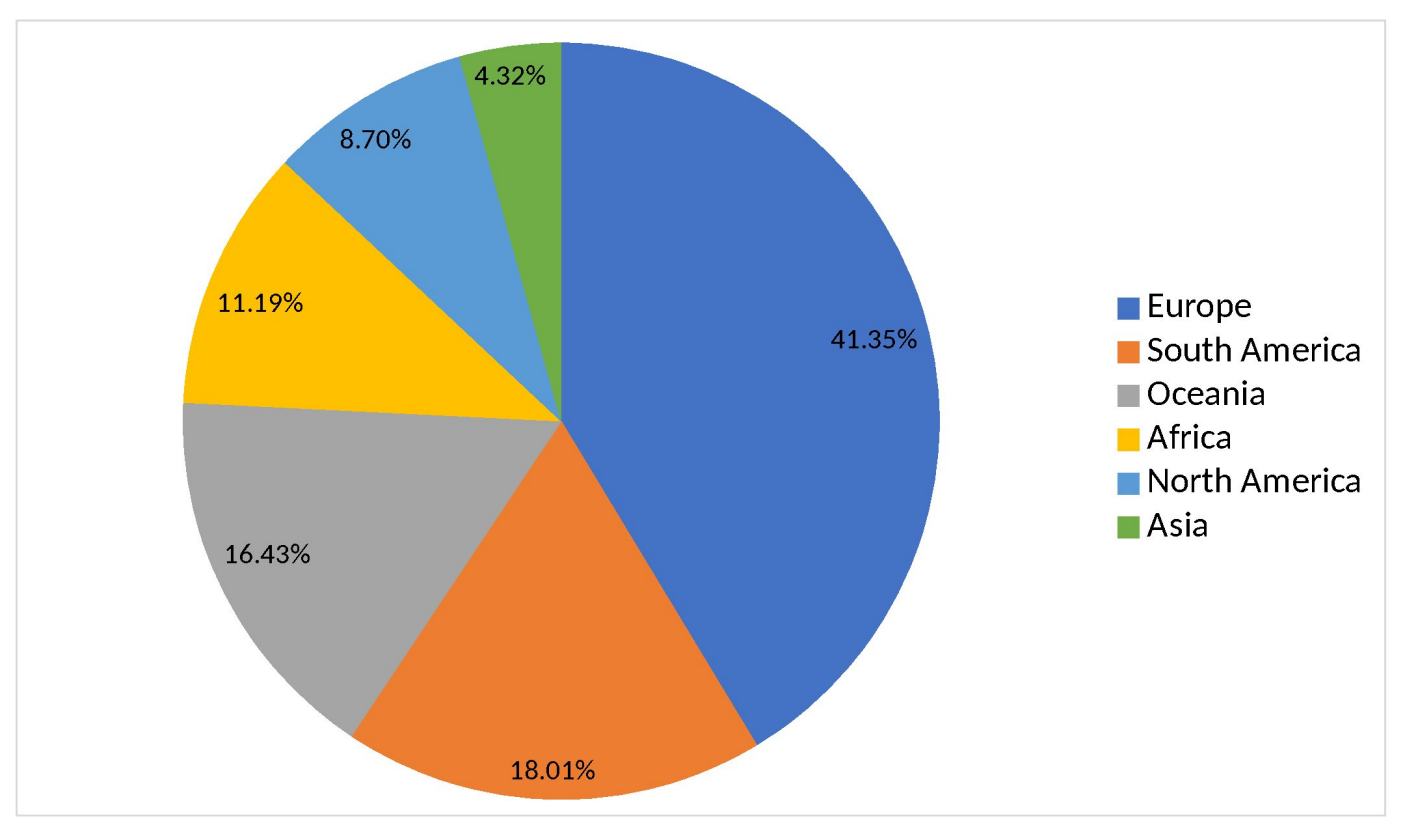
Percentage of introduction records per continent.

**Figure 2.**
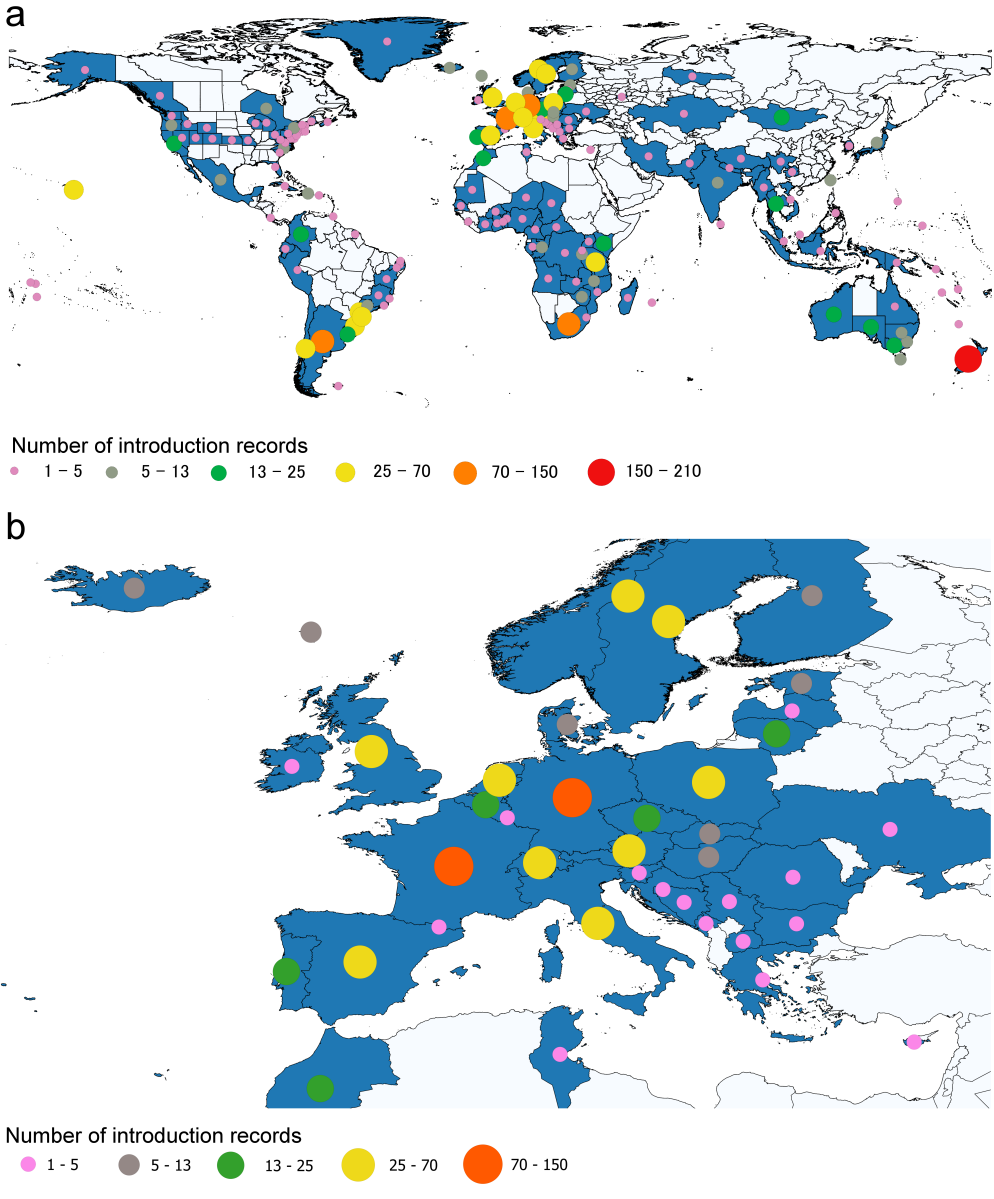
The global (a) and European (b) distribution of the introduced macrofungi. Blue colour represents countries/administrative divisions with at least one introduced species. Circles represent the number of species that have been reported as introduced by both size and colour.

Geographic coverage corresponded to all continental areas, except Antarctica. We collected data from 81 different countries and 61 first-order administrative divisions of the six largest countries. The continent with the highest number of records was Europe (38.78% of records) and the one with the lowest number was Asia (4.7% of records) (Fig. 1). A map showing the number of introduced species per country and administrative divisions, respectively is presented for the world and Europe (Fig. 2). For 26 of the records collected, only the continental-level distribution was possible to assign, as more precise geographical information was unavailable.

## Taxonomic coverage

### Description

**Figure 3.**
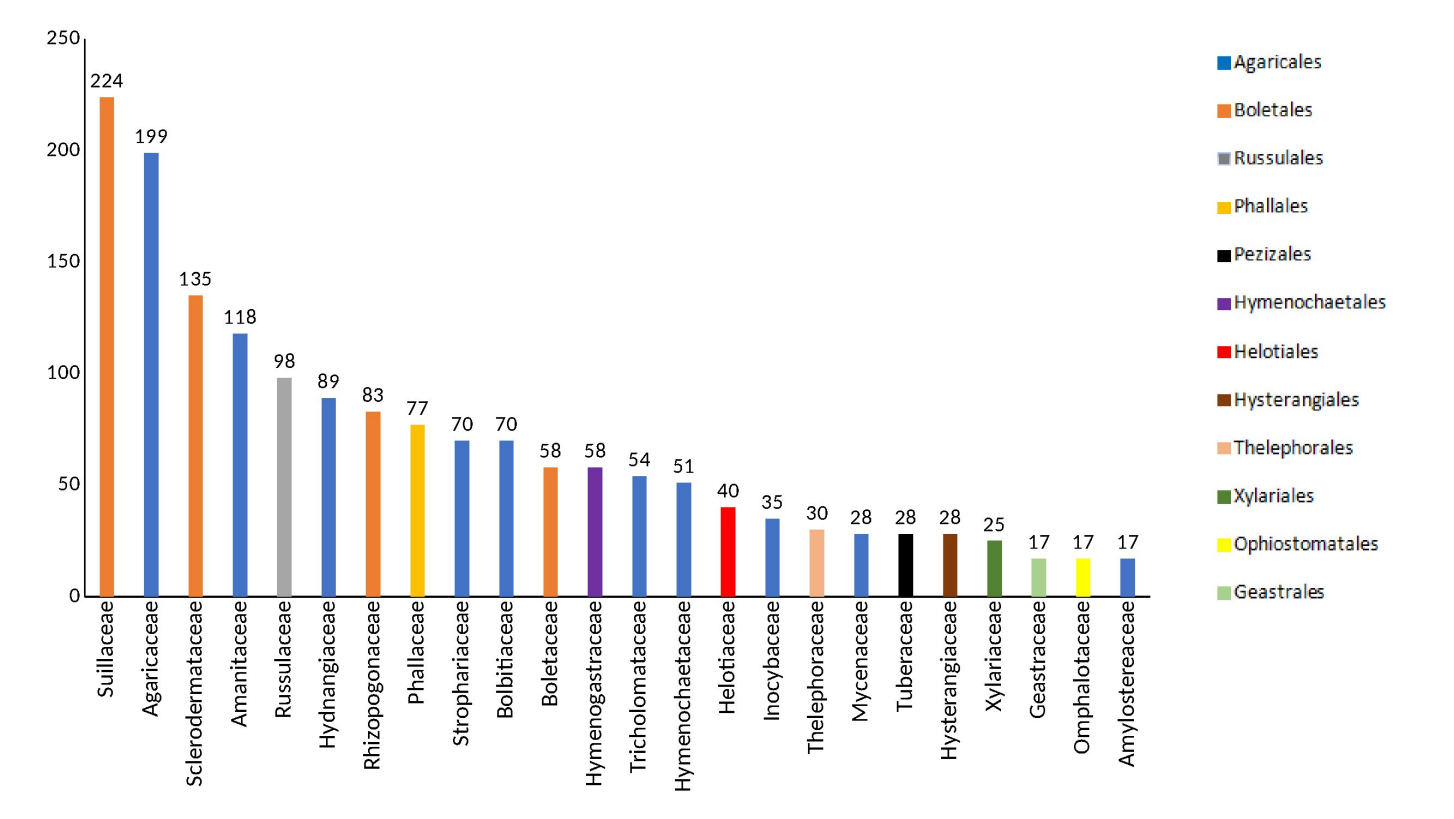
Number of introduction records per family. Only families with more than 17 records are shown. All families were coloured according to their associated order.

The dataset includes distribution records of alien macrofungi taxa from 2 phyla, 7 classes, 22 orders, 82 families, 207 genera, 648 species and 31 varieties, forms or subspecies (Monteiro et al. 2020). Agaricales is the best represented order (44.2% of the records), followed by Boletales (29.2% of records) and Russulales (6.7% of records). The Suillaceae, Agaricaceae and Sclerodermataceae are the families with most alien records (224, 199 and 135 records, respectively) (Fig. 3). Twelve records belong to taxa that were placed *incertae sedis* within their orders as the assignment to a family is yet unclear. Finally, the species with the highest number of alien records are *Suillus luteus* (L.) Roussel (44 records), *Phellinus noxius* (Corner) G. Cunn. (43 records), *Amanita muscaria* (L.) Lam. (38 records), *Amanita phalloides* Secr. (37 records), *Suillus granulatus* (L.) Roussel (34 records) and *Hymenoscyphus fraxineus* (T. Kowalski) Baral, Queloz & Hosoya (34 records).

Two official fungal nomenclatural repositories, Index Fungorum (Index Fungorum 2019) and Mycobank (Robert et al. 2019), were used to resolve taxa and properly attribute the most recent valid names. Index Fungorum was considered our main reference and Mycobank was a secondary resource for some ambiguous cases. Both repositories are currently responsible for documenting the list of scientific names that have been validly defined for fungal taxa.

### Taxa included

**Table:** 

Rank	Scientific Name	
phylum	Basidiomycota	
kingdom	Fungi	
phylum	Ascomycota	
class	Agarocomycetes	
order	Agaricales	
order	Amylocorticiales	
order	Auriculariales	
order	Boletales	
order	Cantharellales	
order	Geastrales	
order	Gloeophyllales	
order	Gomphales	
order	Hymenochaetales	
order	Hysterangiales	
order	Phallales	
order	Polyporales	
order	Russulales	
order	Thelephorales	
class	Dacrymycetes	
order	Dacrymycetales	
class	Tremellomycetes	
order	Tremellales	
class	Dothideomycetes	
order	Pleosporales	
order	Helotiales	
class	Leotiomycetes	
class	Pezizomycetes	
order	Pezizales	
class	Sordariomycetes	
order	Xylariales	

## Temporal coverage

**Data range:** 1785-1-01 – 2018-12-31.

### Notes

**Figure 4.**
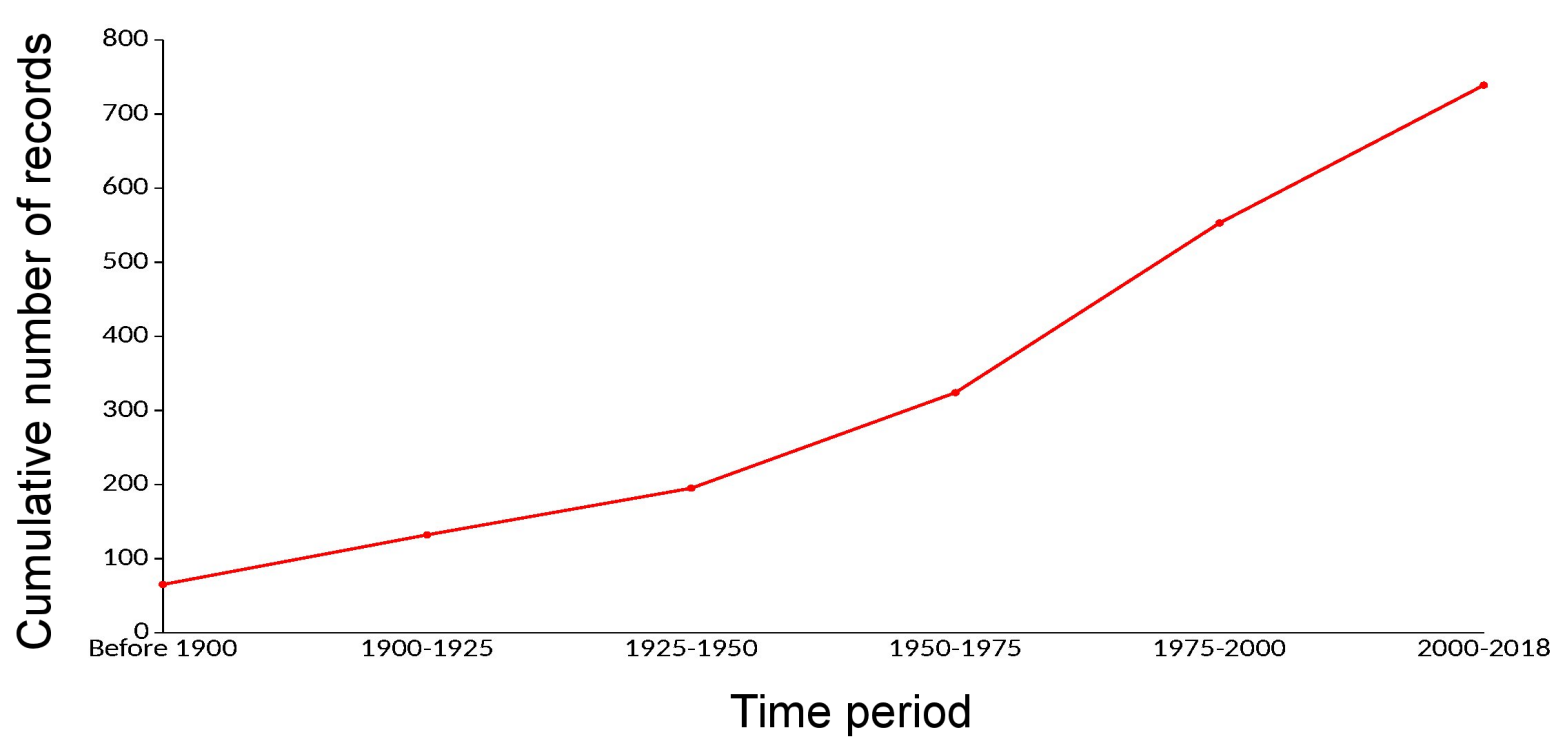
Temporal accumulation of the number of first records in 25‐year periods. The temporal progression is based on 38% of the total of distribution records included in the dataset.

Data sources provided the dates when the species was detected for the first time in a given region for 755 of the 1966 records included in the dataset. The earliest first record dates back to 1753 and the most recent event occurred in 2018. The lowest number of first records is reported between 1900-1925 and the highest number occurred between 1975-2000. Nevertheless, the cumulative number of those introductions grew in a steady way during the referenced period (Fig. 4).

## Usage rights

### Use license

Other

### IP rights notes

CC-BY 4.0

## Data resources

### Data package title

Global database of alien macrofungi

### Resource link

https://www.gbif.org/dataset/da3542b4-9a73-4054-b9a3-2d762e172199

### Alternative identifiers

https://doi.org/10.15468/2qky1q

### Number of data sets

2

### Data set 1.

#### Data set name

Darwin Core Archive Occurrence dataset

#### Data format

Darwin Core Archive format

#### Number of columns

11

#### Character set

UTF-8

#### Download URL

https://www.gbif.org/dataset/da3542b4-9a73-4054-b9a3-2d762e172199

#### Data format version

2.0

#### 

**Data set 1.** 

Column label	Column description
id	Record identifier.
basisOfRecord	The specific nature of the data record.
occurrenceID	Occurrence identifier.
occurrenceRemarks	Occurrence remarks.
establishmentMeans	Establishment means.
associatedReferences	Associated references.
associatedTaxa	Associated taxa.
eventDate	Event date.
countryCode	Country code.
locality	Locality.
taxonID	Taxon identifier.

### Data set 2.

#### Data set name

Darwin Core Archive Taxon dataset

#### Data format

Darwin Core Archive format

#### Number of columns

18

#### Character set

UTF-8

#### Download URL

https://www.gbif.org/dataset/da3542b4-9a73-4054-b9a3-2d762e172199

#### Data format version

2.0

#### 

**Data set 2.** 

Column label	Column description
id	Record identifier.
taxonID	Taxon identifier.
scientificName	The full scientific name, with authorship.
acceptedNameUsage	The full name, with authorship and date information, if known, of the currently valid taxon.
namePublishedIn	A reference for the publication in which the scientificName was originally established under the rules of the associated nomenclaturalCode.
namePublishedInYear	The four-digit year in which scientificName was published.
kingdom	Kingdom name.
phylum	Phylum name.
class	Class name.
order	Order name.
family	Family name.
genus	Genus name.
specificEpithet	Specific epithet.
infraspecificEpithet	Infraspecific epithet.
taxonRank	Taxonomic rank.
scientificNameAuthorship	The authorship information for the scientificName formatted according to the conventions of the applicable nomenclaturalCode.
language	Language of the resource.
datasetName	Dataset name.
